# Glutathione Regulates GPx1 Expression during CA1 Neuronal Death and Clasmatodendrosis in the Rat Hippocampus following Status Epilepticus

**DOI:** 10.3390/antiox11040756

**Published:** 2022-04-11

**Authors:** Ji-Eun Kim, Duk-Shin Lee, Tae-Hyun Kim, Tae-Cheon Kang

**Affiliations:** Department of Anatomy and Neurobiology and Institute of Epilepsy Research, College of Medicine, Hallym University, Chuncheon 24252, Korea; dslee84@hallym.ac.kr (D.-S.L.); hyun1028@hallym.ac.kr (T.-H.K.)

**Keywords:** astrocyte, autophagy, BSO, epilepsy, LAMP-1, NAC, seizure

## Abstract

Glutathione peroxidase-1 (GPx1) catalyze the reduction of H_2_O_2_ by using glutathione (GSH) as a cofactor. However, the profiles of altered GPx1 expression in response to status epilepticus (SE) have not been fully explored. In the present study, GPx1 expression was transiently decreased in dentate granule cells, while it was temporarily enhanced and subsequently reduced in CA1 neurons following SE. GPx1 expression was also transiently declined in CA1 astrocytes (within the stratum radiatum) following SE. However, it was elevated in reactive CA1 astrocytes, but not in clasmatodendritic CA1 astrocytes, in chronic epilepsy rats. Under physiological condition, L-buthionine sulfoximine (BSO, an inducer of GSH depletion) increased GPx1 expression in CA1 neurons but decreased it in CA1 astrocytes. However, N-acetylcysteine (NAC, an inducer of GSH synthesis) did not influence GPx1 expression in these cell populations. Following SE, BSO aggravated CA1 neuronal death, concomitant with reduced GPx1 expression. Further. BSO also lowered GPx1 expression in CA1 astrocytes. NAC effectively prevented neuronal death and GPx1 downregulation in CA1 neurons, and restored GPx1 expression to the control level in CA1 astrocytes. In chronic epilepsy rats, BSO reduced GPx1 intensity and exacerbated clasmatodendritic degeneration in CA1 astrocytes. In contrast, NAC restored GPx1 expression in clasmatodendritic astrocytes and ameliorated this autophagic astroglial death. To the best of our knowledge, our findings report, for the first time, the spatiotemporal profiles of altered GPx1 expression in the rat hippocampus following SE, and suggest GSH-mediated GPx1 regulation, which may affect SE-induced neuronal death and autophagic astroglial degeneration.

## 1. Introduction

The production of hydrogen peroxide (H_2_O_2_) is an essential part of mitochondrial respiration under physiological conditions. Although H_2_O_2_ is not a free radical per se, it forms superoxide (O_2_^•−^) and hydroxyl radical (OH^•^) that cause lipid peroxidation and cell injury. Thus, the endogenously produced hydrogen peroxide has to be removed to prevent oxidative stress. Glutathione (GSH), a tripeptide synthesized from glycine, cysteine, and glutamate, is the most important redox-regulating non-enzymatic thiol that directly scavenges reactive oxygen species (ROS) and acts as a substrate of glutathione peroxidase (GPx) to catalyze the reduction of H_2_O_2_ [1,2].

The GPx family enzymes are found ubiquitously in mammals and eliminate ROS such as hydrogen peroxide and peroxynitrite. GPx isozymes exhibit different tissue-specific expression patterns and subcellular localization: cytosolic GPx (GPx1), gastrointestinal GPx (GPx-GI or GPx2), plasma GPx (GPx-P or GPx3), phospholipid hydroperoxide GPx (PHGPx or GPx4), sperm nuclei GPx (snGPx or GPx5), olfactory GPX (GPx6), GPx7 and GPx8. Among them, GPx1 is the first identified selenoprotein and the most abundant GPx member in the brain, which plays an important role in the antioxidative defense [2,3,4,5]. Indeed, GPx1-depleted astrocytes reduce the H_2_O_2_ clearance rate to cause cell death [6,7], and GPx1 overexpression attenuates cerebral ischemia-reperfusion damage [8].

Epilepsy is a neuronal disorder characterized by recurrent and spontaneous seizures, characterized by excessive, abnormal, and hypersynchronous neuronal activity [9,10,11]. Temporal lobe epilepsy (TLE) is the most common form of partial epilepsy and resistance to anticonvulsive drugs which develops in about 30% of cases [12,13]. Status epilepticus (SE) is an emergency condition showing prolonged and uncontrolled seizure activity, which is one of the epileptogenic factors [14,15,16]. The pilocarpine and lithium (Li)-pilocarpine-induced SE models show the same epileptogenic period: acute (24–48 h), latent (silent) (48 h–3 weeks), and chronic (epilepsy) periods (>3 weeks), and reveal similar hippocampal lesion to those observed in TLE patients [17,18]. In acute and latent periods, massive loss of principal hippocampal (CA1 and CA3) neurons, hilar interneurons, and astrocytes in the molecular layer of the dentate gyrus are observed in the hippocampus [19,20,21]. In the chronic period, CA1 astrocytes show clasmatodendrosis, which is an irreversible autophagic astroglial degeneration [22,23]. Together with devastating plastic changes in the hippocampus vulnerable brain structures [15,16], oxidative stress due to increased formation of ROS contributes to the pathophysiology of SE, subsequent epileptogenic process, and pathogenesis of TLE [24]. Therefore, it is likely that GPx1 would be involved in the injuries of neurons and astrocytes induced by SE, since GPx1 is highly expressed in hippocampal neurons and glial cells [25,26]. However, the altered GPx1 expression in the epileptogenic period has not been fully explored. Furthermore, the role of GPx1 in the pathogenesis of TLE has been controversial. The pilocarpine model shows the transient increase in GPx activity at 1 h after SE [27], while Li-pilocarpine model reveals the reduced GPx activity at the same time window [28]. Unlike ischemic insults [8], GPx1 overexpression increases susceptibility to kainic acid (KA)-induced seizure activity and neuronal hippocampal damage [29]. In addition, both L-buthionine sulfoximine (BSO, an inducer of GSH depletion) and N-acetylcysteine (NAC, an inducer of GSH synthesis) increase or maintain GPx1/2 expression, although GPx1/2 expression depends on the availability of GSH [30,31]. Under circumstances when GSH is deprived, GPx1 can also use γ-glutamylcysteine (a final precursor for GSH synthesis) as a reductant for H_2_O_2_ [32], which would further lead to oxidative stress, and induces ROS-dependent autophagy activation under glucose deprivation conditions by affecting the redox state [33]. Therefore, it is noteworthy to investigate the temporal and spatial alterations in GPx1 expression in the hippocampus and the effects of BSO and NAC on these changes following SE to elucidate the role of GPx1 in the pathegenesis of TLE.

Here, we demonstrate the spatiotemporal alterations in GPx1 expression and the reverse effects of the regulation of GSH level by BSO and NAC on GPx1 expression in neurons and astrocytes in the CA1 regions of the rat hippocampus following SE, which affected SE-induced CA1 neuronal death and clasmatodendritic degeneration of CA1 astrocytes. These findings indicate that GSH consumption led to GPx1 downregulation, which subsequently resulted in CA1 neuronal death and autophagic astroglial degeneration induced by SE and epileptic seizures. Therefore, our findings highlight the importance of the preservation/maintenance of GSH levels in the protection of neurons and astrocytes from detrimental stress thereby increasing GPx1 expression.

## 2. Materials and Methods

### 2.1. Experimental Animals and Chemicals

We used adult male Sprague-Dawley (SD) rats (7 weeks old) in the present study. Animals were kept under standard environmental conditions (23–25 °C, 12 h light/dark cycle) and freely accessed to water and food in the experiments. All experimental protocols were conducted with the approval of the Institutional Animal Care and Use Committee of Hallym University (Hallym 2021-30, approval date: 17 May 2021). All reagents were obtained from Sigma-Aldrich (St. Louis, MO, USA), except as noted.

### 2.2. SE Induction and Chronic Epilepsy Model

In the present study, we used the LiCl-pilocarpine model of temporal lobe epilepsy (TLE): One day before pilocarpine injection, animals were treated LiCl (127 mg/kg, i.p.). Then, 20 min before pilocarpine administration, atropine methylbromide (5 mg/kg i.p.) was injected. Thereafter, rats were treated with pilocarpine (30 mg/kg, i.p.). Two hours after status epilepticus (SE) onset, diazepam (Valium; Hoffmann-la Roche, Neuilly-sur-Seine, France; 10 mg/kg, i.p.) was administered to control seizure activity and repeated, as needed. Control animals received saline in place of pilocarpine. Animals were video-monitored 8 h a day to determine chronic epileptic rats showing spontaneous seizures [34,35,36].

### 2.3. BSO and NAC Treatment

One day after SE, vehicle, L-buthionine-sulfoximine (BSO, 1.3 g/kg) or N-acetylcysteine (NAC, 70 mg/kg) was administered once a day by intraperitoneal (i.p.) over 2 days. In our previous studies [37,38], these dosages of BSO and NAC effectively affected the brain’s GSH contents. Some control and chronic epilepsy animals were also administered vehicle, BSO, or NAC by the same methods. Five hours after the last injection, animals were used for immunohistochemistry.

### 2.4. Western Blot

Animals were anesthetized with urethane anesthesia (1.5 g/kg, i.p.) and decapitated. The hippocampus was quickly removed and homogenized in lysis buffer. The concentration of total protein was determined using a Micro BCA Protein Assay Kit (Pierce Chemical, Dallas, TX, USA). Thereafter, Western blot was performed by the standard protocol (*n* = 7 in each group). Briefly, after electrophoresis, membranes were incubated with the primary antibodies (Table 1) and protein bands were detected and quantified on an ImageQuant LAS4000 system (GE Healthcare Korea, Seoul, Korea). β-Actin was used as an internal reference to normalize the number of proteins [34,35,36].

### 2.5. Immunohistochemistry

Animals were anesthetized with urethane anesthesia (1.5 g/kg, i.p.) and underwent transcardiac perfusion with normal saline followed by 4% paraformaldehyde in 0.1 M phosphate buffer (PB, pH 7.4). Afterward, brains were processed with post-fixation in the same fixative overnight. Brain tissues were cryoprotected by infiltration with 30% sucrose overnight followed by cryosection at 30 μm. After 3-time washing with PBS (0.1 M, pH 7.3), sections were pre-blocked with 3% bovine serum albumin in PBS for 30 min at room temperature and incubated with a cocktail solution containing primary antibodies (Table 1) in PBS containing 0.3% Triton X-100 overnight at room temperature. Thereafter, sections were reacted with appropriate Cy2-, Cy3-, or aminomethylcoumarin acetate (AMCA)-conjugated secondary antibodies. A negative control test was performed with preimmune serum substituted for the primary antibody. Experimental procedures in this study were carried out under the same conditions and in parallel. To measure fluorescent intensity, the random-selected 5 areas/animals (300 μm^2^/area) in the hippocampus (5 sections from each animal, *n* = 7 in each group) and fluorescent intensity was measured by using AxioVision Rel. 4.8 and ImageJ software. Fluorescent intensity was represented as the number of a 256-gray scale after standardization with mean background intensity obtained from 5 image inputs. Manipulation of the images was restricted to threshold and brightness adjustments to the whole image. For quantification of clasmatodendritc astrocytes, sections (10 sections per each animal) were captured and cell counts in areas of interest (1 × 10^4^ μm^2^) were selected from the stratum radiatum of the CA1 region were performed using AxioVision Rel. 4.8 Software [34,35,36].

### 2.6. Data Analysis

The data were expressed as mean ± standard error of the mean. Comparisons between groups were performed using Student *t*-test or one-way analysis of variance (ANOVA) followed by Newman–Keuls post-hoc test. The *p* < 0.05 was considered to be statistically significant.

## 3. Results

### 3.1. SE Changes Hippocampal GPx1 Expression in a Regional Specific Manner

**Figure 1:**
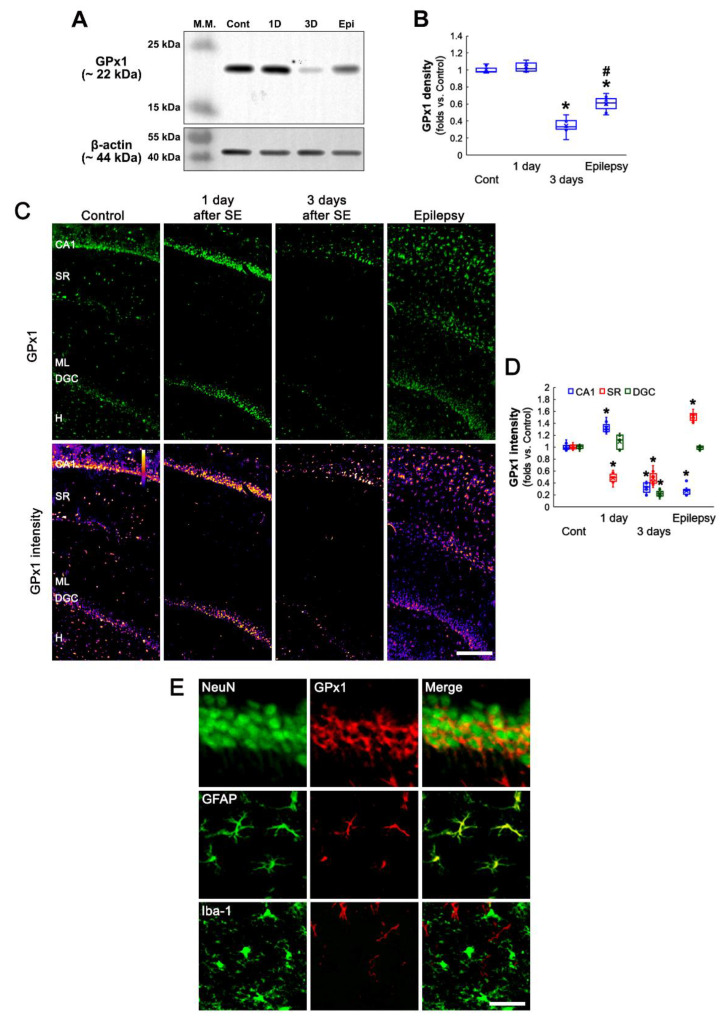
Effects of SE on GPx1 expression in the rat hippocampus. GPx1 expression is mainly observed in neurons (NeuN, a neuronal marker) and astrocytes (GFAP, an astroglial marker), but not microglia (Iba-1, a microglial marker), under physiological conditions. Following SE, GPx1 expression shows the temporal and spatial alterations in the hippocampus. (**A**) Representative Western blot of GPx1 in the whole hippocampus. (**B**) Quantification of GPx1 protein level based on Western blot data (*,^#^ *p* < 0.05 vs. control and 3 day-post SE animals, *n* = 7, respectively). (**C**) Representative photos of GPx1 expression and its intensity. Bar = 250 μm. (**D**) Quantification of GPx1 intensity in CA1 neurons (CA1), the stratum radiatum (SR) and dentate granule cells (DGC) (* *p* < 0.05 vs. control animals, *n* = 7, respectively). (**E**) Cellular localization of GPx1 in the hippocampus under physiological conditions. Bar = 25 (NeuN) and 12.5 μm (GFAP and Iba-1).

Western blot data revealed that SE did not affect GPx1 expression level in the hippocampus at 1 day after SE. However, GPx1 expression was reduced at 3 days after SE. In chronic epilepsy rats, GPx1 expression was increased as compared to 3-day post-SE animals, although it was lower than that in control animals (*F*_(3,24)_ = 137.27, *p* < 0.001, one-way ANOVA, *n* = 7, respectively; Figure 1A,B and Supplementary Figure S1). Immunohistochemical studies also demonstrated that SE-induced alterations in GPx1 expression showed a regional-specific pattern in the hippocampus (Figure 1C,D). Consistent with previous studies [25,26], GPx1 expression was obviously observed in hippocampal neurons and glial cells in control animals (Figure 1C). One day after SE, GPx1 expression level was increased in CA1 neurons (*t*_(12)_ = 7.03, *p* < 0.001, Student *t*-test, *n* = 7, respectively; Figure 1C,D), while it significantly reduced in glial cells within the stratum radiatum (*t*_(12)_ = 12.83, *p* < 0.001, Student *t*-test, *n* = 7, respectively; Figure 1C,D). The SE did not affect GPx1 level in dentate granule cells (DGC, Figure 1C,D). Three days after SE, GPx1 expression was markedly diminished in CA1 neurons (*t*_(12)_ = 14.97, *p* < 0.001, Student *t*-test, *n* = 7, respectively; Figure 1C,D) and DGC (*t*_(12)_ = 26.87, *p* < 0.001, Student *t*-test, *n* = 7, respectively; Figure 1C,D). In chronic epilepsy rats, GPx1 expression was elevated in glial cells within the stratum radiatum more than control animals (*t*_(12)_ = 13.82, *p* < 0.001, Student *t*-test, *n* = 7, respectively; Figure 1C,D), and was restored to control level in DGC (Figure 1C,D). A double immunofluorescent study identified that GPx1 expression was mainly observed in neurons and astrocytes, but not microglia, under physiological conditions (Figure 1E). These findings indicate that the altered GPx1 expression would be related to the distinct responses of neurons and astrocytes to SE, which would be masked in the Western blot study.

### 3.2. Altered GPx1 Expression Is Relevant to Neuronal Vulnerability to SE

**Figure 2:**
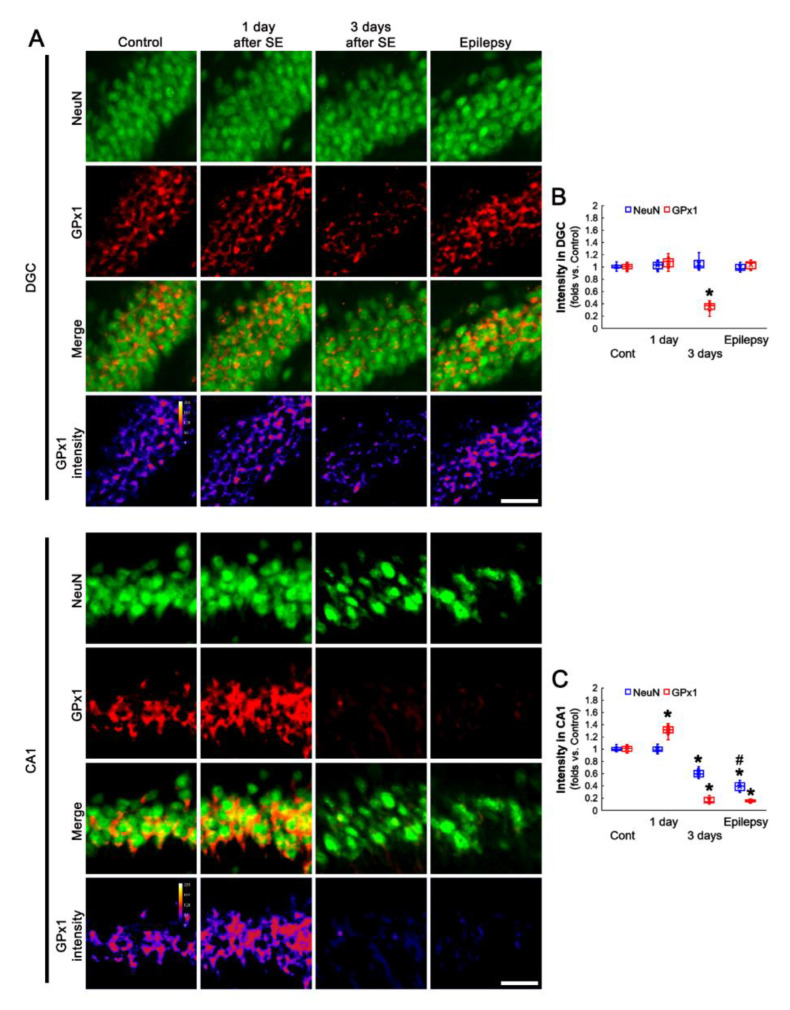
Neuronal GPx1 expression in dentate granule cells (DGC) and CA1 neurons following SE. In DGC, GPx1 expression is transiently reduced at 3 days after SE. In CA1 neurons, however, GPx1 expression is temporally increased at 1 day after SE, and gradually reduced, accompanied by neuronal loss. (**A**) Representative photos of GPx1 expression and its intensity. Bar = 25 μm. (**B**,**C**) Quantification of GPx1 intensity in DGC (**B**) and CA1 neurons (**C**) (*,^#^ *p* < 0.05 vs. control and 3 day-post SE animals, *n* = 7, respectively).

The vulnerability of hippocampal neurons shows the regional specific heterogenicity in response to SE: DGC are remarkably resistant to most insults, while CA1 neurons are extremely vulnerable to various stimuli [39,40,41]. Therefore, we investigated the temporal and spatial alterations in GPx1 expression following SE to elucidate the relevance between GPx1 and neuronal vulnerability. In DGC, NeuN expression was unaffected by SE. However, GPx1 expression was transiently decreased at 3 days after SE, which was restored in chronic epilepsy rats (*F*_(3,24)_ = 132.84, *p* < 0.001, one-way ANOVA, *n* = 7, respectively; Figure 2A,B). However, NeuN expression was gradually diminished in CA1 neurons at 3 days after SE indicating neuronal death (*F*_(3,24)_ = 241.1, *p* < 0.001, one-way ANOVA, *n* = 7, respectively; Figure 2A,C). GPx1 expression in CA1 neurons was temporarily enhanced at 1 day after SE, and subsequently reduced in 3 day-post-SE and chronic epilepsy rats (*F*_(3,24)_ = 685.64, *p* < 0.001, one-way ANOVA, *n* = 7, respectively; Figure 2A,C). These findings indicate that the preservation or restoration of GPx1 may be involved in the vulnerability of hippocampal neurons to SE.

### 3.3. BSO and NAC Differently Affect GPx1 Expression in CA1 Neurons under Physiological and Post-SE Conditions

**Figure 3:**
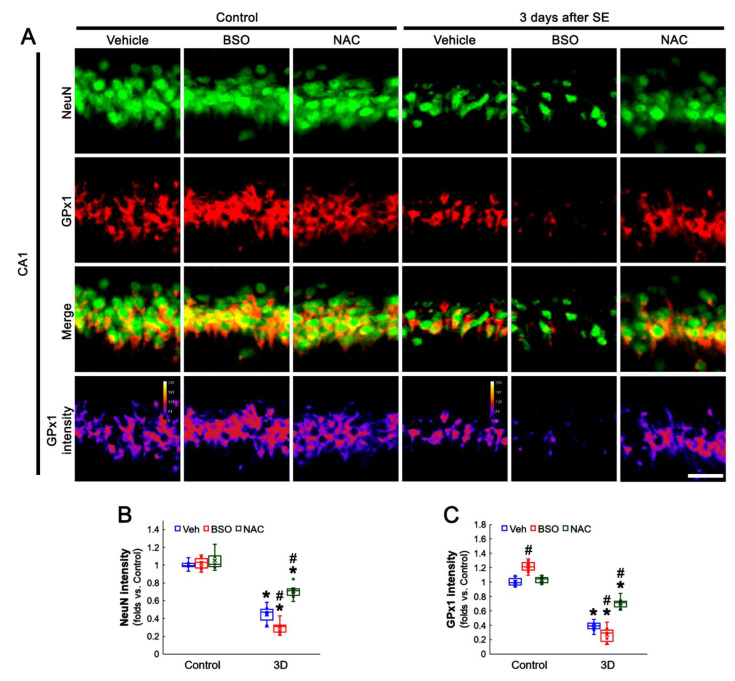
Effects of BSO and NAC on GPx1 expression in CA1 neurons under physiological- and 3 day-post-SE conditions. Under physiological conditions, BSO increases GPx1 expression, while NAC does not affect it, as compared to vehicle (Veh). Three days after SE, BSO aggravates SE-induced CA1 neuronal death accompanied by the reduced GPx1 expression. NAC attenuates SE-induced CA1 neuronal loss and decreased GPx1 expression. (**A**) Representative photos of GPx1 expression and its intensity. Bar = 25 μm. (**B**,**C**) Quantification of NeuN (**B**) and GPx1 (**C**) intensity in CA1 neurons (**C**) (*,^#^ *p* < 0.05 vs. control and vehicle, *n* = 7, respectively).

**Figure 4:**
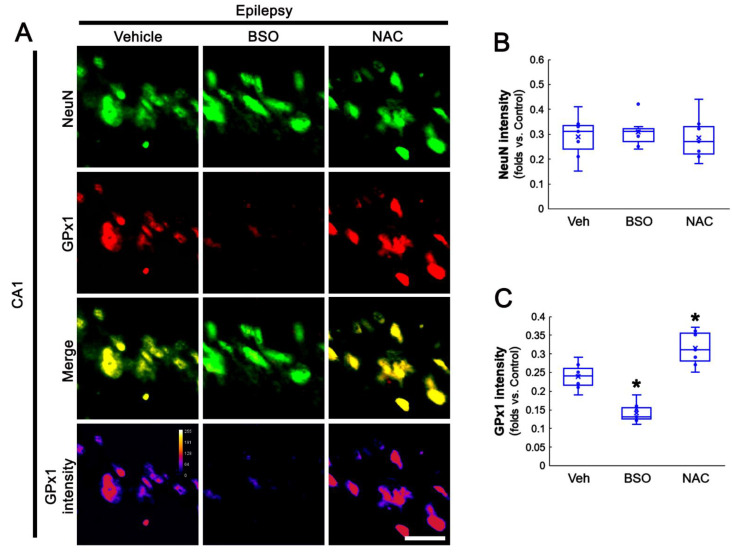
Effects of BSO and NAC on GPx1 expression in CA1 neurons of chronic epilepsy rats. As compared to vehicle (Veh), BSO diminishes GPx1 expression, while NAC increases it, without affecting NeuN intensity. (**A**) Representative photos of GPx1 expression and its intensity. Bar = 25 μm. (**B**,**C**) Quantification of NeuN (**B**) and GPx1 (**C**) intensity in CA1 neurons (**C**) (* *p* < 0.05 vs. control and vehicle, *n* = 7, respectively).

Since pilocarpine reduces the total GSH level in the rat hippocampus within the acute and chronic (epilepsy) stages [42,43], it is likely that the alterations in GPx1 expression may be adaptive responses to reduced GSH levels induced by acute and chronic seizure activity. To confirm the relationship between GSH level and GPx1 expression, we applied BSO and NAC in animals under physiological and post-SE conditions. This is because BSO leads to GSH depletion by inhibiting GSH synthesis and NAC should fuel GSH synthesis as its precursor [44,45]. As compared to vehicles, both BSO and NAC did not affect NeuN expression in CA1 neurons of control animals (Figure 3A,B). However, BSO increased GPx1 expression in CA1 neurons of control animals (*t*_(12)_ = 5.53, *p* < 0.001, Student *t*-test, *n* = 7, respectively), while NAC did not influence it (Figure 3A,C). Three days after SE (two days after treatment), BSO aggravated SE-induced CA1 neuronal death (*t*_(12)_ = 3.07, *p* = 0.005, Student *t*-test, *n* = 7, respectively; Figure 3A,B), concomitant with the reduced GPx1 expression (*t*_(12)_ = 2.33, *p* = 0.04, Student *t*-test, *n* = 7, respectively; Figure 3A,C). In contrast, NAC effectively prevented neuronal death induced by SE (*F*_(2,18)_ = 39.9, *p* < 0.001, one-way ANOVA, *n* = 7, respectively; Figure 3A,B). The NAC also attenuated the reduced GPx1 expression in CA1 neurons (*F*_(2,18)_ = 45.73, *p* < 0.001, one-way ANOVA, *n* = 7, respectively; Figure 3A,C). In chronic epilepsy rats, BSO and NAC did not influence NeuN intensity in CA1 neurons (Figure 4A,B). As compared to vehicle, BSO diminished GPx1 expression in CA1 neurons (*t*_(12)_ = 5.8, *p* < 0.001, Student *t*-test, *n* = 7, respectively), while NAC increased it (*t*_(12)_ = 3.43, *p* = 0.002, Student *t*-test, *n* = 7, respectively; Figure 4A,C). These findings indicate that BSO and NAC may distinctly affect GPx1 expression in CA1 neurons under physiological and post-SE conditions and that altered GPx1 expression may be a result of GSH depletion induced by SE. In addition, our findings reveal that GSH consumption in CA1 neurons may be higher than that in DGC following SE.

### 3.4. SE Leads to the Biphasic Alterations in GPx1 Expression in CA1 Astrocytes

**Figure 5:**
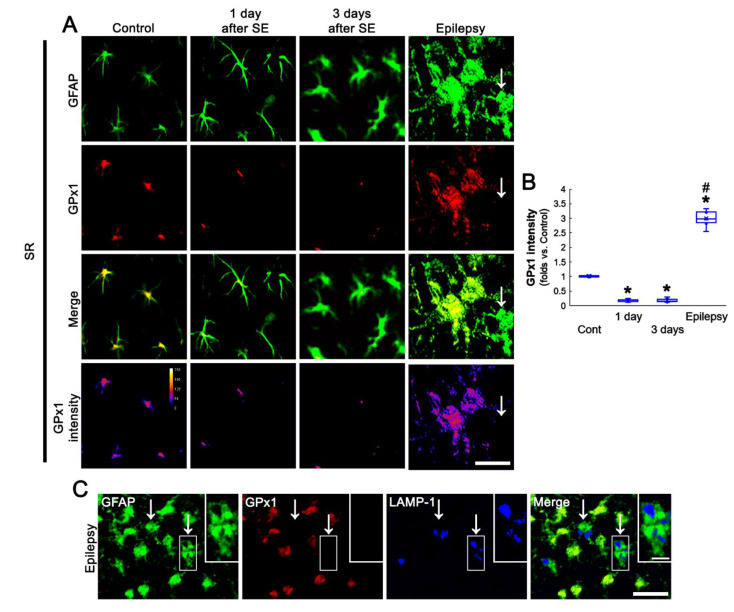
Astroglial GPx1 expression in the stratum radiatum (SR) of the CA1 region following SE. GPx1 expression is reduced at 1 day and 3 days after SE, but upregulated in reactive CA1 astrocytes of chronic epilepsy rats. GPx1 expression is rarely detected in clasmatodendritic CA1 astrocytes (arrows). (**A**) Representative photos of GPx1 expression and its intensity. Bar = 12.5 μm. (**B**) Quantification of GPx1 intensity in CA1 astrocytes (*,^#^ *p* < 0.05 vs. control and 3 day-post SE animals, *n* = 7, respectively). (**C**) Representative photos of clasmatodendritic CA1 astrocytes containing lysosome-associated membrane protein 1 (LAMP1)-positive vacuoles (arrows). Insertions are high magnification of rectangles. Bar = 25 and 12.5 μm (insertion).

Next, we also explored the relationship between GPx1 expression and astroglial responses to SE. One day after SE, CA1 astrocytes possessed the processes showing unevenly thick, as compared to naive astrocytes (Figure 5A). Three days SE, CA1 astrocytes had hypertrophic cell bodies and processes (Figure 5A). GPx1 expression was declined in CA1 astrocytes at 1 day and 3 days after SE. In chronic epilepsy rats, GPx1 expression was significantly elevated in CA1 astrocytes more than control animals (*F*_(3,24)_ = 583.16, *p* < 0.001, one-way ANOVA, *n* = 7, respectively; Figure 5A,B). In chronic epilepsy rats, CA1 astrocytes showed typical reactive astrogliosis (hypertrophy and hyperplasia of cell bodies and processes). In addition, some CA1 astrocytes had round-shaped and edematous cell bodies, short blunt processes, GFAP tangles, and lysosome-associated membrane protein 1 (LAMP-1)-positive vacuoles, indicating clasmatodendritic degeneration (Figure 5A,C). GPx1 expression was upregulated in reactive CA1 astrocytes, while it was rarely observed in clasmatodendritic CA1 astrocytes (Figure 5A,C).

### 3.5. BSO Enhances, but NAC Abrogates SE-Induced GPx1 Downregulation in CA1 Astrocytes

**Figure 6:**
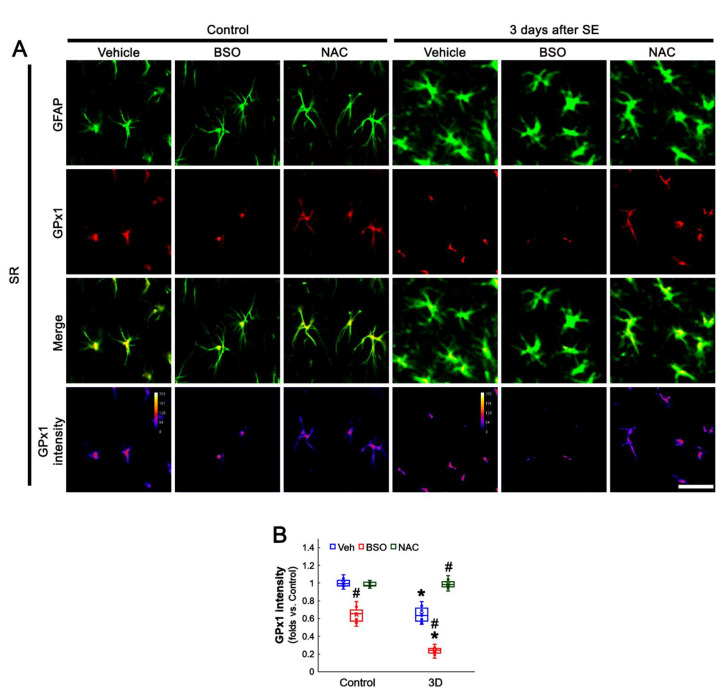
Effects of BSO and NAC on GPx1 expression in the stratum radiatum (SR) of the CA1 region under physiological- and 1 day-post-SE condition. Under physiological conditions, BSO reduces GPx1 expression, while NAC does not affect it, as compared to vehicle (Veh). Three days after SE, BSO more decreases GPx1 expression. NAC restores GPx1 expression to the control level. (**A**) Representative photos of GPx1 expression and its intensity. Bar = 25 μm. (**B**) Quantification of GPx1 (**B**) intensity in CA1 astrocytes (*,^#^ *p* < 0.05 vs. control and vehicle, *n* = 7, respectively).

Since the present data showed neuronal GPx1 expression responded differently to BSO and NAC treatment, we also explored the effects of BSO and NAC on astroglial GPx1 expression. Under physiological condition, BSO decreased GPx1 expression in CA1 astrocytes, as compared to vehicle (*t*_(12)_ = 8.42, *p* < 0.001, Student *t*-test, *n* = 7, respectively), while NAC did not affect it (Figure 6A,B). Three days after SE (two days after treatment), BSO lowered GPx1 expression in CA1 astrocytes, as compared to vehicle (*t*_(12)_ = 9.81, *p* = 0.005, Student *t*-test, *n* = 7, respectively; Figure 6A,B). However, NAC restored GPx1 expression to control level (*t*_(12)_ = 7.95, *p* < 0.001, Student *t*-test, *n* = 7, respectively; Figure 6A,B). These findings indicate that BSO may reduce GPx1 expression in CA1 astrocytes, while NAC may preserve it, under post-SE conditions.

### 3.6. BSO Exacerbates, but NAC Attenuates Clasmatodendrosis in CA1 Astrocytes of Chronic Epilepsy Rats

**Figure 7:**
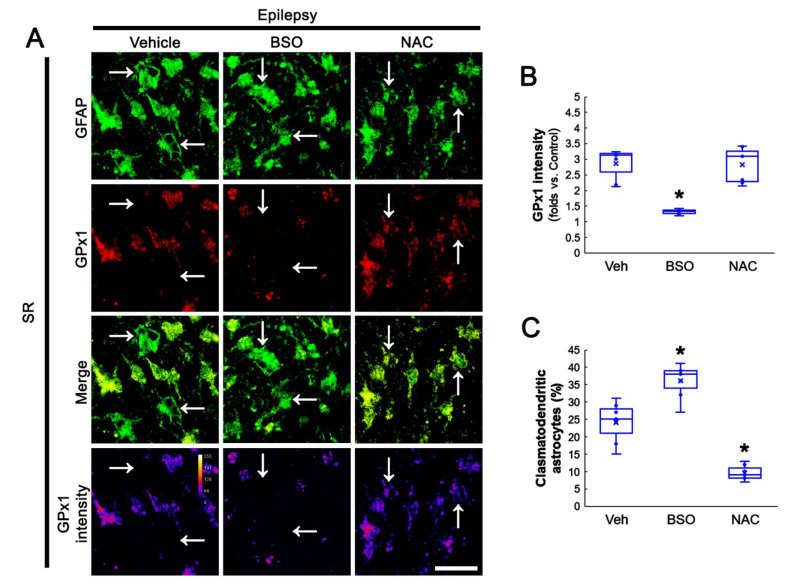
Effects of BSO and NAC on GPx1 expression in the stratum radiatum (SR) of the CA1 region of chronic epilepsy rats. As compared to vehicle (Veh), BSO diminishes GPx1 expression and exacerbates clasmatodendrosis of CA1 astrocytes (arrows). NAC does not affect GPx1 intensity in CA1 astrocytes, but ameliorates this autophagic astroglial degeneration with increased GPx1 intensity in vacuolized astrocytes. (**A**) Representative photos of GPx1 expression and its intensity. Bar = 25 μm. (**B**,**C**) Quantification of GPx1 (**B**) intensity and clasmatodendritic degeneration (**C**) in CA1 astrocytes (* *p* < 0.05 vs. vehicle, *n* = 7, respectively).

Oxidative stress is one of the major causes leading to clasmatodendrosis [36,46]. Therefore, we also validated the effects of BSO and NAC on clasmatodendrosis in the hippocampi of chronic epilepsy rats. As compared to vehicle, BSO reduced GPx1 intensity, while NAC did not affect it, in CA1 astrocytes (*F*_(2,18)_ = 28.92, *p* < 0.001, *n* = 7, respectively, one-way ANOVA; Figure 7A,B). However, GPx1 expression was detected in some vacuolized CA1 astrocytes (Figure 7A). Furthermore, BSO aggravated clasmatodendritic degeneration, but NAC ameliorated it, in CA1 astrocytes (*F*_(2,18)_ = 58.95, *p* < 0.001, *n* = 7, respectively, one-way ANOVA; Figure 7A,C). Considering NAC-induced preservation of GPx1 expression in clasmatodendritic CA1 astrocytes, our findings indicate that GPx1 upregulation in CA1 astrocytes may be an adaptive response against oxidative stress induced by GSH deficiency and may delay or prevent autophagic astroglial degeneration in the epileptic hippocampus.

## 4. Discussion

The major findings in the present study are that decreased GPx1 expression did not represent a neuronal vulnerability to SE. The regulation of GSH levels by BSO or NAC differently affected GPx1 expression in CA1 neurons and CA1 astrocytes under physiological and post-SE conditions. Furthermore, GPx1 upregulation in CA1 astrocytes was an adaptive response against oxidative stress, and reduced GPx1 expression led to clasmatodendritic degeneration of CA1 astrocytes in the epileptic hippocampus. Therefore, our findings indicate that GSH is the primary antioxidant central to the regulations of cell responses and GPx1 level towards oxidative stress induced by seizure activity, and that the maintenance of GPx1 level may prevent autophagic astroglial degeneration.

The hippocampal neurons show the heterogeneous vulnerability in response to harmful stresses: DGC are remarkably resistant, but CA1-, CA3 neurons, and dentate hilar neurons are extremely vulnerable [39,47]. In previous studies, we found that the underlying mechanisms of SE-induced neuronal death are relevant to oxidative stress, but distinct from each neuronal population: SE-induced oxidative stress leads to aberrant mitochondrial elongation leading to necrosis of CA1 neurons, and to excessive mitochondrial fragmentation resulting in apoptosis of hilar interneurons [40,48,49]. Indeed, Li-pilocarpine-induced SE increases H_2_O_2_ production in the hippocampus during the acute period and returned to control levels during the latent period, and then significantly increased again during the chronic period [50]. Therefore, we had speculated that the dysregulation of GPx1 expression would be involved in the differential vulnerability of hippocampus neurons in response to SE.

In the present study, we found that SE increased GPx1 expression in CA1 neurons, but not DGC, at 1 day after SE. However, GPx1 expression was markedly diminished in CA1 neurons and DGC 3 days after SE. Furthermore, GPx1 expression was restored to the control level in DGC in chronic epilepsy rats, while it was decreased in CA1 neurons accompanied by massive neuronal loss. Considering that the total GSH level in the hippocampus is decreased at the acute and chronic (epilepsy) stages of the rat pilocarpine epilepsy model [42,43], it is presumable that reduced GSH level would affect the differential GPx1 expression in the hippocampal neurons following SE. Indeed, the present study reveals that BSO increased GPx1 expression in CA1 neurons without inducing neuronal death under physiological conditions. BSO leads to GSH depletion by selectively inhibiting γ-glutamylsysteine synthase (the first step enzyme of GSH synthesis) [44]. Compatible with the present data, BSO-induced GSH depletion results in GPx1/2 upregulation caused by high amounts of H_2_O_2_ [30,31]. Therefore, it is likely that the transient GPx1 upregulation in CA1 neurons 1 day after SE may be an adaptive response against oxidative stress induced by SE-induced GSH depletion. In the present study, however, BSO exacerbated SE-induced CA1 neuronal death with reduced GPx1 expression 3 days after SE. BSO also diminished GPx1 expression in CA1 neurons of chronic epilepsy rats. Since BSO deteriorates GPx1/2 downregulation in response to 2-hydroxyethyl methacrylate-induced oxidative stress [30,31] and oxidative stress leads to decreased GSH level followed by GPx1 degradation [33], our findings indicate that the enhanced oxidative stress induced by sustained GSH consumption may lead to GPx1 degradation in CA1 neurons and DGC at 3 days after SE when the amount of H_2_O_2_ would be increased far beyond the capacity of GPx1. NAC, a derivate of cysteine, fuels GSH synthesis under stress conditions. In addition, NAC per se is an intracellular source of sulfhydryl groups and acts as a direct ROS scavenger [45]. In the present study, NAC effectively attenuated CA1 neuronal death and the reduced GPx1 expression 3 days after SE, although it did not affect GPx1 intensity under physiological conditions. Furthermore, NAC increased GPx1 expression in CA1 neurons of chronic epilepsy rats. GPx1 predominates in the elimination of low concentrations of H_2_O_2_ since at higher H_2_O_2_ concentrations the GPx1 reaction rate reaches a ceiling due to the limited NADPH and GSH supplies [51]. Indeed, NAC constantly keeps GSH amounts at high levels leading to increased GPx1/2 expression [30,31]. With respect to these previous studies, it is likely that transient GPx1 upregulation could not cope with the prolonged GSH consumption in CA1 neurons in response to SE-induced oxidative stress. Under this condition, the sustained oxidative stress due to GSH depletion might subsequently lead to GPx1 degradation in CA1 neurons, and in turn, a decreased GPx1 capacity for defending against ROS would contribute to CA1 neuronal death. In contrast to CA1 neurons, GPx1 expression in DGC was unaltered in 1-day post-SE and chronic epilepsy animals, although it was transiently diminished 3 days after SE. These findings indicate that GSH synthesis would rapidly recover in DGC. Therefore, our findings support the idea that altered GPx1 expression may be a result of the difference in the degree of GSH consumption, and suggest that the regulation of GPx1 expression affected by capacity of restoring GSH synthesis in each neuronal population may determine the differential vulnerability to oxidative stress between CA1 neurons and DGC.

GPx1 is important for the antioxidative defense of brain cells since neurons and astrocytes cultured from GPx1 knockout mice are more vulnerable than wild-type cells to the toxicity of H_2_O_2_ [4,6,52]. However, neuronal antioxidant activity is not sufficiently high to decompose H_2_O_2_ generated by themselves. Thus, neurons are protected by glial cells with high GPx1 content in the brain [53]. Indeed, astrocytes play a key role in cellular defense mechanisms against oxidative stress [54]. In the present study, altered GPx1 expression showed the biphasic pattern in CA1 astrocytes following SE: GPx1 expression was decreased in CA1 astrocytes at 1 day and 3 days after SE, which was restored to the control level by NAC treatment. Furthermore, BSO declined GPx1 expression under physiological- and post-SE conditions. These findings indicate that astroglial GPx1 expression may be also regulated by GSH level. Since astrocytes are also one of the sources of the H_2_O_2_ and redox imbalance following SE [50], furthermore, it is likely that GPx1 downregulation in CA1 astrocytes may contribute to CA1 neuronal death by eliciting oxidative stress, which was aggravated by BSO treatment. In the present study, GPx1 expression was elevated in reactive CA1 astrocytes in chronic epilepsy rats more than in control animals. Similar to the present data, GPx expression is increased in glial cells surrounding the surviving dopaminergic neurons in the substantia nigra of patients with Parkinson’s disease. Furthermore, increased GPx expression is correlated with the severity of dopaminergic cell loss [53]. Therefore, it is likely that GPx1 upregulation in reactive CA1 astrocytes may be a compensatory response contributing to protecting remaining CA1 neurons against oxidative stress induced by repeated spontaneous seizures.

Clasmatodendrosis is an irreversible astroglial injury characterized by extensive swollen cell bodies with vacuoles and disintegrated/beaded processes, which is first reported by Alzheimer, and later Cajal termed it “clasmatodendrosis” [55,56]. We have first reported that clasmatodendrosis is autophagic astroglial degeneration since vacuoles in clasmatodendritic astrocytes are identified as active lysosomes [22]. Other investigators also confirmed that various pathological conditions lead to clasmatodendrosis by activating autophagocytosis and the ubiquitin proteasome system [57,58]. Thus, clasmatodendrosis is an autophagic astroglial degeneration as non-apoptotic (type II) programmed cell death, independent of caspase activity [59]. In the epileptic hippocampus, clasmatodendritic changes in CA1 astrocytes affect seizure duration and are further deteriorated by spontaneous seizures [36,46,60]. Although the underlying mechanisms are largely unknown, oxidative stress is one of the major causes leading to clasmatodendrosis [36,46]. Unlike reactive CA1 astrocytes, the present study reveals that GPx1 expression was rarely observed in clasmatodendritic CA1 astrocytes. Furthermore, BSO exacerbated clasmatodendritic degeneration of CA1 astrocytes concomitant with reduced GPx1 intensity, while NAC ameliorated this autophagic astroglial degeneration and increased GPx1 expression in some vacuolized CA1 astrocytes. Therefore, our findings indicate that prolonged GSH consumption induced by oxidative stress may downregulate GPx1 expression, and subsequently lead to clasmatodendrosis in CA1 astrocytes. In addition, preservation of GPx1 expression may delay or prevent autophagic astroglial degeneration of CA1 astrocytes.

On the other hand, GPx1 overexpression increases seizure susceptibility and neuronal hippocampal damage in response to KA, since alterations in the redox state of the GSH system result in elevated oxidized GSH (GSSG) levels, which directly reinforce N-methyl-D-aspartate (NMDA) receptor functionality [29]. Regarding the role of clasmatodendrosis in epileptic seizures [36,46,60] and the effect of NAC on clasmatodendrosis and GPx1 expression in the present study, however, it is unlikely that GPx1 upregulation in reactive CA1 astrocytes would exert ictogenesis (seizure generation) via NMDA receptor activation in the epileptic hippocampus.

There is little in the literature concerning the profiles of GPx1 protein levels and its activity in TLE patients. However, Yüzbaşioğlu et al. [61] have reported that GPx1 mRNA is upregulated in the hippocampal tissues obtained from TLE patients, and suggested that this upregulation may be a result of a shift in the proportion of neuronal and glial cell populations due to the severe neuronal loss in samples. Consistent with this report, the present data demonstrate the GPx1 upregulation in reactive CA1 astrocytes. Therefore, it is likely that the increased GPx1 expression in astrocytes may result in its upregulation in the hippocampus of TLE patients.

## 5. Conclusions

The present study reveals for the first time the temporal and spatial alterations in GPx1 expression following SE, which were regulated by the degree of GSH consumption. Furthermore, GPx1 downregulation in CA1 astrocytes elicited clasmatodendritic degeneration. Therefore, our findings highlight the importance of the preservation/maintenance of GSH levels in the protection of neurons and astrocytes from detrimental stress induced by SE and epileptic seizures, thereby increasing GPx1 expression.

## Figures and Tables

**Table 1:** Primary antibodies used in the present study.

Antigen	Host	Manufacturer (Catalog Number)	Dilution Used
GPx1	Sheep	Biosensis (#S-072-100)	1:10,000 (WB) 1:2000 (IH)
NeuN	Guinea pig	Millipore (#ABN90P)	1:1000 (IH)
LAMP-1	Rabbit	Abcam (#ab24170)	1:100 (IH)
GFAP	Mouse	Millipore (#MAB3402)	1:2000 (IH)
Iba-1	Rabbit	Biocare Medical (#CP 290)	1:500 (IH)
β-actin	Mouse	Sigma (#A5316)	1:5000 (WB)

IH: Immunohistochemistry; WB: Western blot.

## Data Availability

Not applicable.

## Supplementary Materials

The following supporting information can be downloaded at: https://www.mdpi.com/article/10.3390/antiox11040756/s1, Figure S1: Full-length gel images of Western blot data in Figure 1A.
