# Supplementary material for: Glutathione Regulates GPx1 Expression during CA1 Neuronal Death and Clasmatodendrosis in the Rat Hippocampus following Status Epilepticus
Source: Antioxidants (Basel). 2022 Apr 11;11(4):756. doi: 10.3390/antiox11040756 (PMC9024994; doi:10.3390/antiox11040756)
Supplement: Supplementary file 1 [file antioxidants-11-00756-s001.zip › antioxidants-1637686-supplementary.pdf]

Supplementary information

Glutathione regulates GPx1 expression during CA1 neuronal death and clasmatodendrosis in the rat hippocampus following status epilepticus

Ji-Eun Kim[1]*, Duk-Shin Lee[1] , Tae-Hyun Kim[1] and Tae-Cheon Kang [1,]*

[1] Department of Anatomy and Neurobiology and Institute of Epilepsy Research, College of Medicine, Hallym University, Chuncheon 24252, Korea; dslee84@hallym.ac.kr (D-SL), hyun1028@hallym.ac.kr (T.-H.K.).

***** Correspondence: jieunkim@hallym.ac.kr; Tel: +82-33-248-2524; Fax: +82-33-248-2525 and tckang@hallym.ac.kr; Tel: +82-33-248-2524; Fax: +82-33-248-2525.

Fig. 1A

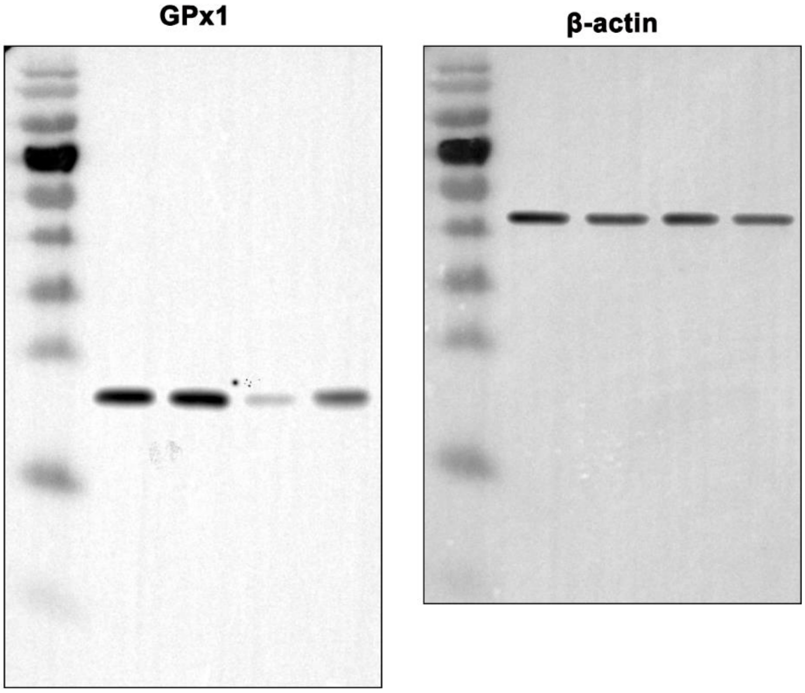

Supplementary Figure S1. Full-length gel images of Western blot data in Figure 1A.
